# Accuracy of radiographic projections to guide cephalic screw position in pertrochanteric fracture: a cadaveric study

**DOI:** 10.1007/s00590-023-03690-z

**Published:** 2023-08-25

**Authors:** Francesco Lazzarini, Tommaso Paoli, Andrea Cozzi Lepri, Gregorio Secci, Luigi Zanna, Matteo Innocenti, Fabrizio Matassi, Christian Carulli, Roberto Civinini

**Affiliations:** 1 Orthopaedic Clinic, University of Florence, Careggi University Hospital, Largo Palagi 1, 50139 Florence, Italy; 2 Department of Orthopedic Surgery, Santa Maria Annunziata Hospital,, Via Antella 58, 50012 Bagno a Ripoli, Italy

**Keywords:** Proximal femur, Pertrochanteric fractures, Cadaveric study, Löwenstein lateral view, True lateral view, Screw positioning

## Abstract

**Purpose:**

The aim of this study was to evaluate the relationship between the Löwenstein Lateral view and the True Lateral view for the positioning of the cephalic hip screw, through a cadaveric study.

**Materials and Methods:**

We placed two Kirschner wires in eight femur specimens using an Antero-Posterior view, Löwenstein Lateral view and True Lateral view. The distances between the Kirschner wires and the anterior, posterior, superior and inferior cortex were measured in all projections. The head of the femur was then sectioned, and the same macroscopic distances were measured. Finally, we could calculate the accuracy of the two radiographic lateral projections.

**Results:**

When the Kirschner wire was placed in the center of the head using the Antero-Posterior and the True Lateral view, the accuracy of Antero-Posterior view was 0.9705 while the accuracy of True Lateral view and Löwenstein Lateral view was 1.1479 and 1.1584, respectively. When the Kirschner wire was placed superior on the Antero-Posterior and centrally on the True Lateral view, the accuracy of Antero-Posterior view was 0.9930 while the accuracy of True Lateral view and Löwenstein Lateral view was 1.1159 and 0.7224, respectively.

**Conclusion:**

When the Kirschner wire was positioned proximal in Antero-Posterior view and central in True Lateral view, only the True Lateral view showed high accuracy.

## Introduction

Intraoperative fluoroscopy has been increasingly essential in trauma surgery [1]. Using a mobile C-arm fluoroscopy virtually infinite projections can be obtained by adjusting the inclination of the intensifier. However, it is not always easy to understand images and choose the proper position of the C-arm to have a realistic view of the bony segment [2].

Nowadays, hip fracture is considered a major healthcare problem, with a 1-year mortality up to 30% [3, 4] and increasing incidence, due to the aging of the general population [5]. Pertrochanteric fracture is a common fracture and the most popular treatment options are cephalomedullary nail and sliding hip screw-plate [6, 7]. Both techniques require the correct position of lag screw through the neck and the head in antero-posterior (AP) view and axial view of the hip [8]. An incorrect positioning of the lag screw is predictive of the implant failure [9, 10], especially considering the Tip-Apex Distance [8, 11] and the Cleveland zones [12]. Despite improvements in systems and techniques of proximal femur fixation, the failure rate remains up to 16% [13].

In order to provide the correct position of the lag screw, two types of lateral views are described: the ﻿Löwenstein lateral view (LLV), which is defined as a lateral view with the C-arm placed horizontally, and the so called true lateral view (TLV), which is defined as a lateral view with the C-arm inclined approximately 15–20° from the coronal plane, parallel to the femoral neck anteversion [14]. However, in most cases, it is the surgeon’s choice to establish which is the best projection to guide the surgery, even if LLV, which is easier to reproduce, is the most common used.

To our knowledge there are no studies to date that have compared the two lateral views to guide correct position of the lag screw in Proximal Femur. The purpose of this study is to investigate the relationship between the LLV and the TLV for the positioning of the cephalic hip screw, using cadaveric femurs. We hypothesize that TLV allows to identify the real position of the lag screw in the femoral head, guiding the surgeon to a better evaluation of implant position.

## Material and methods

### Specimens

We conducted our study using eight femurs (5 right, and 3 left) from 8 fresh frozen cadavers (4 males, and 4 females, selected without bone pathologies). The mean age was 87.5 years (range, 77–94 years; standard deviation (SD) 6.16).

The femurs were cleaned from the surrounding soft tissues. Main proximal femur geometric parameters of our specimens were reported: major head diameter, neck-shaft angle, and anteversion angle.

### Technical procedure

**Fig. 1:**
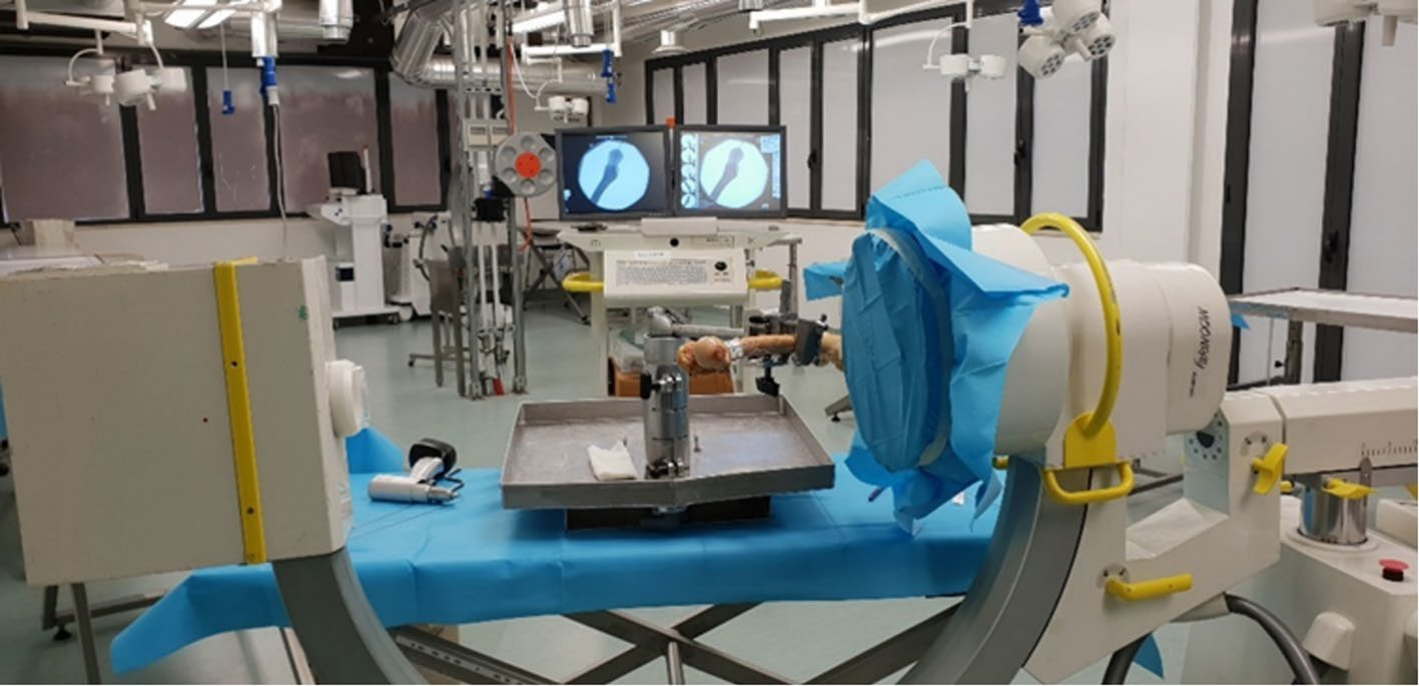
The image illustrate the position of femur and image intensifier, positioned simulating the real intraoperative orientation

Each femur was clamped horizontally and facing upwards on a rigid support with the femoral trochlea parallel to the ground, to simulate intraoperative position of the patient on the traction table. The C-arm was also positioned simulating the intraoperative orientation (Fig. 1).

Under fluoroscopic guidance, we placed two Kirschner (K) wires (with a diameter of 2 mm) at various femoral head and neck heights.

The K-wires insertion and evaluation was made through a standard AP projection, a LLV and a TLV.

AP projection was obtained by positioning the X-ray tube of the C-arm perpendicular to the ground floor.

LLV was obtained by turning the image intensifier horizontally and so with the X-ray tube parallel to the ground floor.

**Fig. 2:**
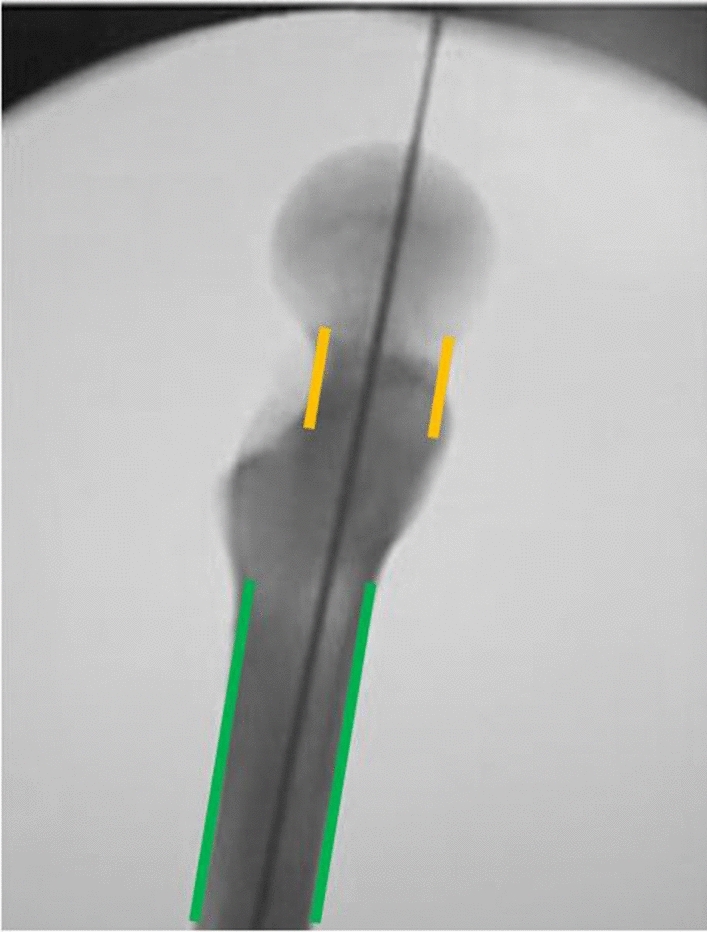
True Lateral View (TLV) obtained when the anterior and posterior cortex of the neck (yellow lines) are perfectly parallel to anterior and posterior cortex of the shaft (green lines) (color figure online)

The TLV was obtained by positioning the C-arm following physiological anteversion of the femoral neck, to align the axis of the neck with the axis of the shaft on the same plane and have a perfect parallelism between head-neck and the shaft. The axis of the neck and the axis of the shaft were considered parallel assessing the anterior and posterior cortex of the neck and the shaft. When they were parallel, neck and shaft were considered as aligned (Fig. 2).

**Fig. 3:**
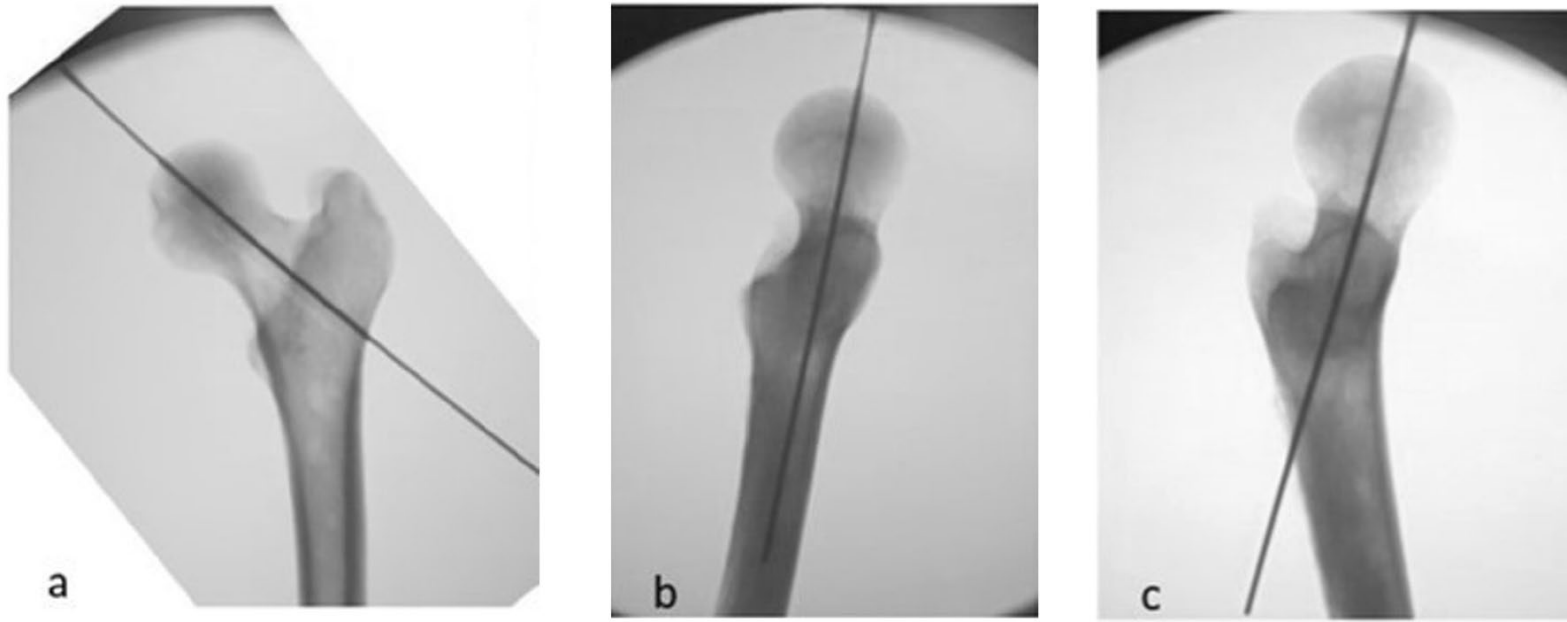
**a** Kirschner (K) wire placed into the center of the neck and femoral head in Antero-Posterior projections. **b** K-wire placed as much as possible into the center of the neck and femoral head in True Lateral view (TLV), paying attention to the parallelism between K-wire and femoral diaphysis. **c** Löwenstein Lateral view (LLV) performed after positioning K-wire to verify a possible change of position of the K-wire on head and femur neck

A first K-wire (K1) was placed as much as possible into the center of the neck and femoral head in both the TLV and the AP projections, paying attention to the parallelism between K-wire and femoral diaphysis on the TLV. A LLV was then performed to verify a possible modification of the K-wire position (Fig. 3).

**Fig. 4:**
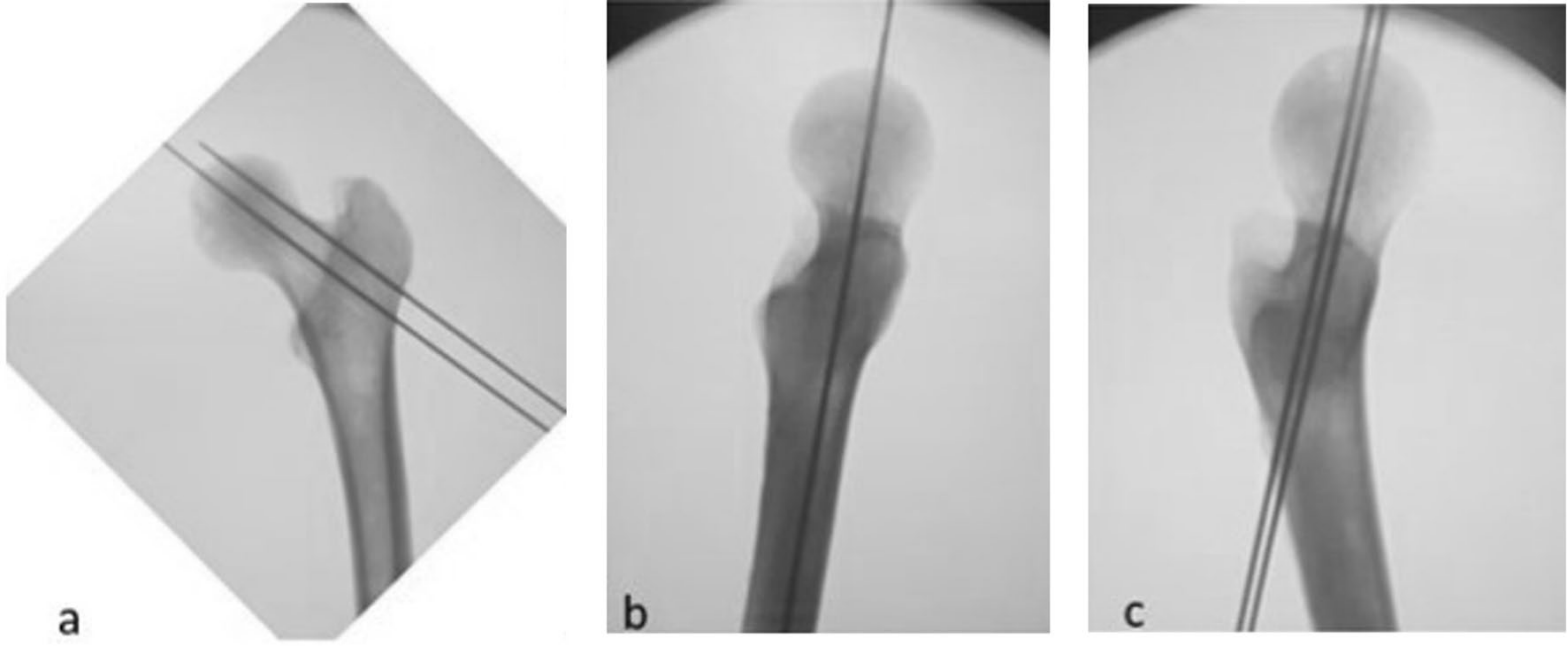
**a** Second Kirschner (K) wire positioned proximal compared to the previous and parallel to the previous one in Antero-Posterior view. **b** Second K-wire positioned as much as possible parallel to the previous one in True Lateral view (TLV).** c** Löwenstein Lateral view (LLV) performed after positioning second K-wire to verify a possible change of position of the second K-wire on femoral head-neck

Using the TLV and the AP projection, a second K-wire (K2) were positioned proximal compared to the previous one in AP view and parallel to the previous one in both projections (AP and TLV). The parallelism of both K-wires with the femoral shaft was maintained in TLV as much as possible. A LLV was then performed to verify a possible modification of the K-wires position (Fig. 4).

On the AP view and on each of the two lateral projections (LLV and TLV), through the Brainlab—TraumaCad® software, the position of K-wire into the femoral head was measured. All measurements were calibrated based on the known K-wire diameter. Through the software we normalize femoral head measure on X-rays by circles passing through the superior, the inferior and the medial extreme of the femoral head cortex in AP view and passing through the apex, the anterior extremity, and the extreme posterior of the femoral head cortex in both lateral projections (LLV, TLV). We drew two lines perpendicular to the K-wires (K1 and K2) and passing through the center of the circumference obtained from the normalized femoral head, thus measuring the distance between the K-wires (K1 and K2) and the superior cortex and inferior cortex on the AP view and between the K-wires (K1 and K2) and the anterior cortex and posterior cortex on the two lateral views (Figs. 6, 7, 8).

**Fig. 5:**
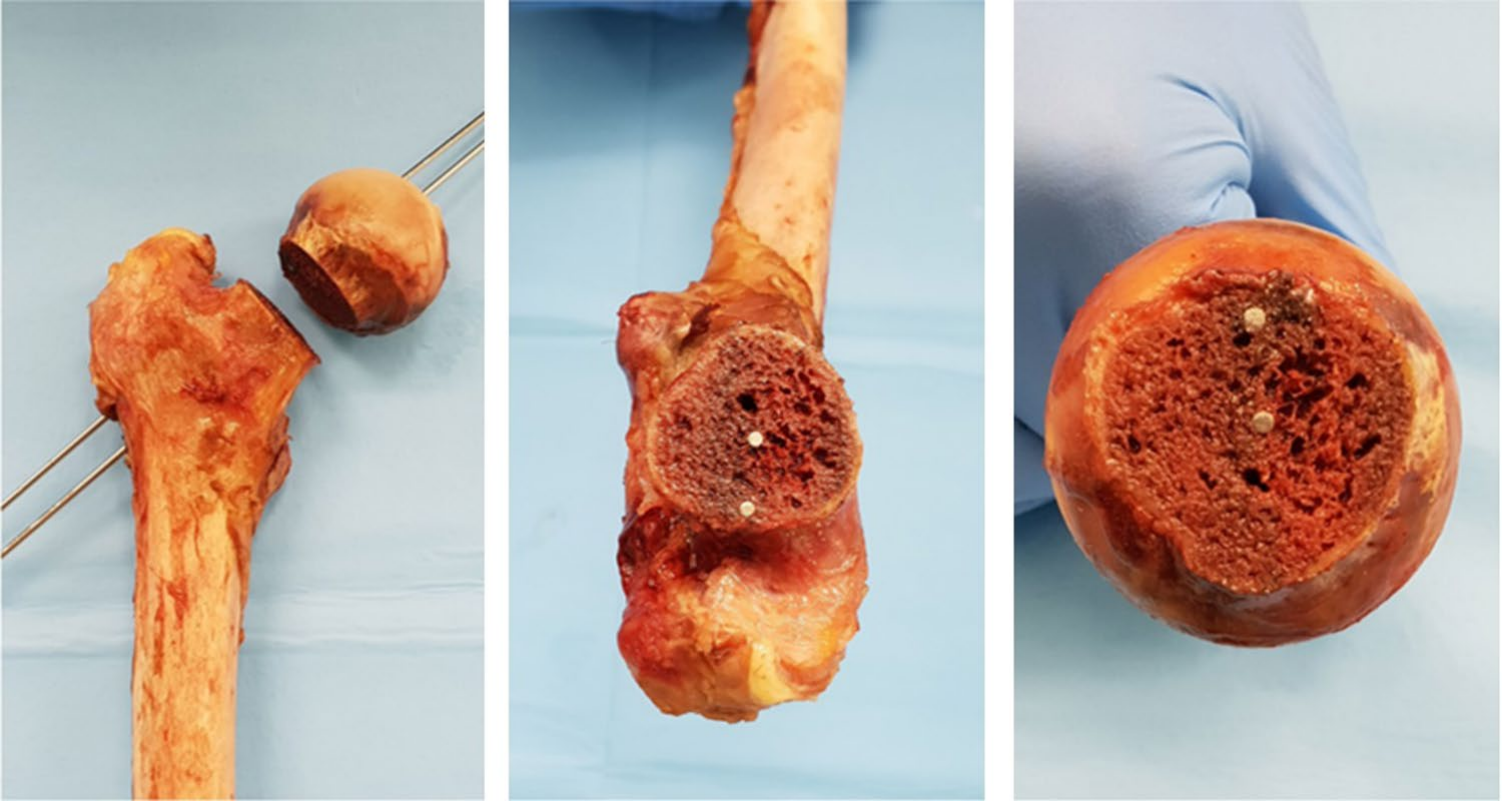
Macroscopic image of sawn femur at the base of the head after the final positioning of Kirschner wires

After the definitive placement of K-wires femur was sawed at the base of the femoral head and macroscopic position of wires was collected and documented with photographs (Fig. 5).

The position of the K-wire was then measured to record the real macroscopic localization.

On the image of the dissected femoral head, through the Brainlab—TraumaCad® software we normalize femoral head measure on photographs by circles passing through the superior cortex, the anterior extremity, and the extreme posterior of the femoral head cortex, we drew two lines perpendicular to the K-wires (K1 and K2) and to the diameter of the circumference obtained from the normalized femoral head. Then we measured the distance between the two K-wires (K1 and K2) and the superior cortex, inferior cortex, anterior cortex, and posterior cortex (Fig. 9).

Finally, we calculated the accuracy of all measurements that is the expression of how much a measure is close to the real value. To do this we used the following formula:

$${\text{Accuracy }}\left( A \right) = \frac{{{\text{True }}\;{\text{value}}}}{{{\text{Measured}}\;{\text{ value}}}}$$

We consider those obtained by macroscopic images of the dissected femoral head as *True values* and those obtained by fluoroscopic images as *Measured values*. We used as True and Measured values the respective ratios between the anterior/posterior and superior/inferior distances. The highest accuracy is to be considered for a value equal to 1; for this we calculated the absolute difference between 1 (Δ) and the various measured accuracy results.

### Ethical approval

This research was approved by the ethical committee of our Institution.

## Results

The average major head diameter of the femur was 46.75 mm ± 3.693 (SD), the average neck-shaft angle was 131.125° ± 1.885 (SD) and the average anteversion angle was 17° ± 1.560 (SD) (Table 1).

**Table 1:** Main proximal femur geometric parameters of the specimens were reported: the average Femoral Head diameter (FHD), Neck-Shaft Angle (NSA), Anteversion Angle (AA)

	Specimen anatomy	
	Average	SD
FHD	46.75 mm	3.693
NSA	131.125°	1.885
AA	17°	1.560

The TLV was obtained by tilting the C-arm relative to the ground following the anteversion angle of each femur.

**Fig. 6:**
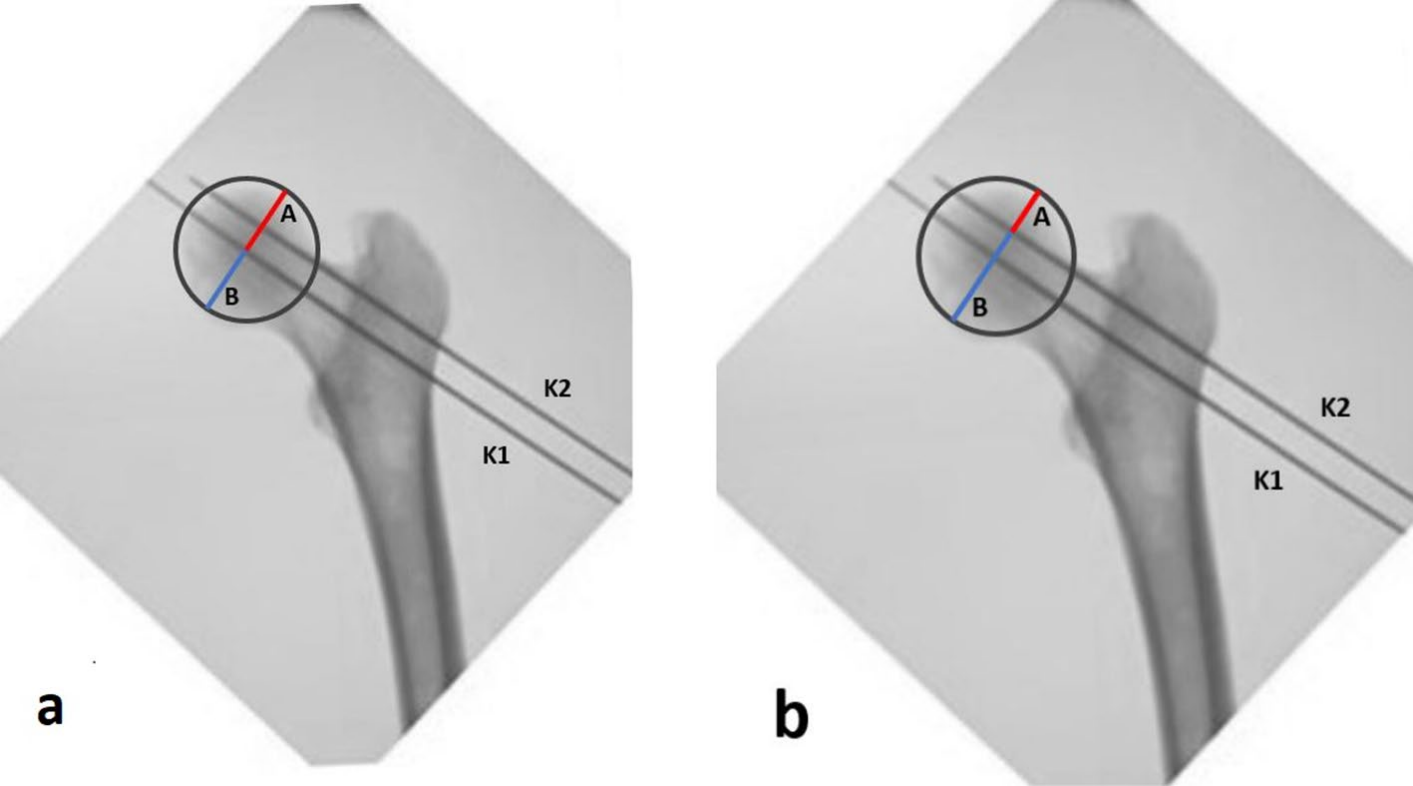
Through TraumaCad® software on Antero-Posterior view femoral head was normalized by circles passing through the superior, the inferior and the medial extreme of the femoral head cortex. **a** We drew two lines perpendicular to the first Kirschner wire (K1) and passing through the center of the circumference obtained from the normalized femoral head, thus measuring the distance between K1 and the superior cortex (A) and the distance between K1 and inferior cortex (B).** b** We drew two lines perpendicular to the second Kirschner wire (K2) and passing through the center of the circumference obtained from the normalized femoral head, thus measuring the distance between K2 and the superior cortex (A) and the distance between K2 and inferior cortex (B)

On the AP view the mean distance between the K1 wire and the superior cortex was 24.212 ± 1.940 mm, while the mean distance between the K1 wire and the inferior cortex was 25.800 ± 1.445 mm, determining a mean ratio of 0.9416 ± 0.097 (Fig. 6a).

On the AP view the mean distance of K2 wire from the superior femoral head cortex was 12.112 ± 1.758 mm, while the mean value from the inferior cortex was 37.912 ± 1.734 mm, mean ratio 0.3186 ± 0.035 (Fig. 6b).

**Fig. 7:**
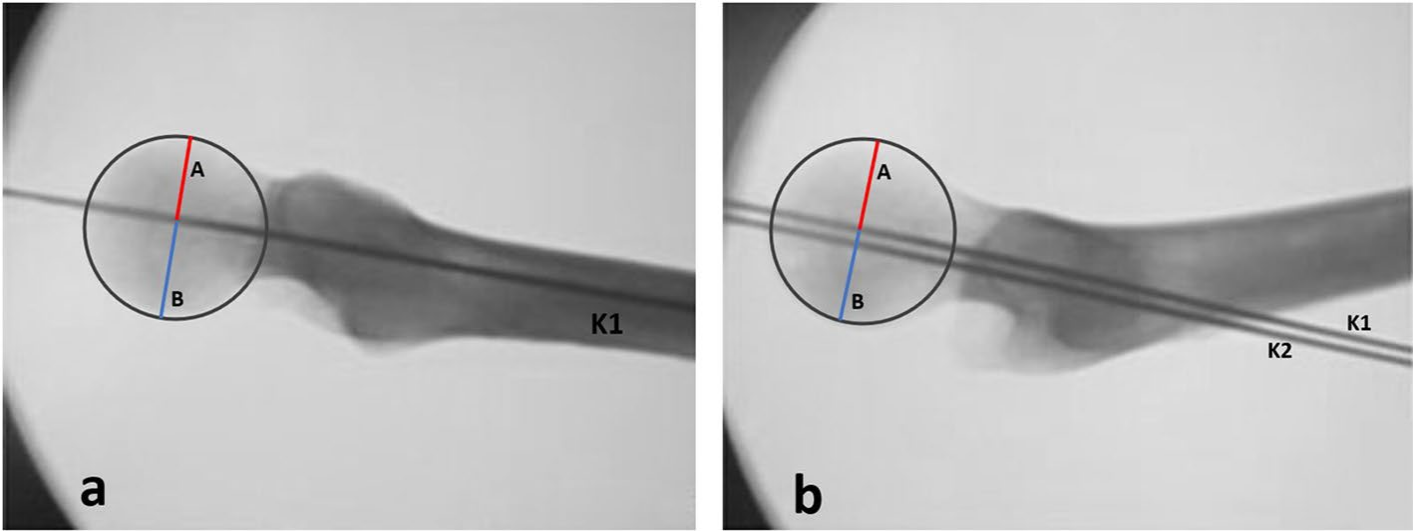
Through Brainlab-TraumaCad® software image femoral head was normalized by circles passing through the apex, the anterior extremity and the extreme posterior of the femoral head cortex in both Löwenstein Lateral view (LLV) and True Lateral view (TLV). **a** On the image obtained through the TLV, K1 and K2 are superimposed; we drew two lines perpendicular to the first Kirschner (K1) and passing through the center of the circumference obtained from the normalized femoral head, thus measuring the distance between K1 and the anterior cortex (A) and the distance between K1 and posterior cortex (B).** b** On the image obtained through the LLV we drew two lines perpendicular to the K1 and passing through the center of the circumference obtained from the normalized femoral head, thus measuring the distance between K1 and the anterior cortex (A) and the distance between K1 and posterior cortex (B)

On the TLV the mean distance between the K1 wire and the anterior cortex was 16.825 ± 1.7539 mm and the mean distance between the K1 wire, and the posterior cortex was 20.79 ± 1.032 mm, mean ratio 0.8105 ± 0.088 (Fig. 7a).

On the LLV the mean distance between the K1 wire and the anterior cortex of the femoral head was 14.700 ± 1.592 mm and a mean distance between K1 wire, and the posterior cortex was 18.312 ± 1.295 mm, ratio 0.8032 ± 0.0722 (Fig. 7b).

On the TLV the mean distance between the K2 wire and the anterior cortex was 16.837 ± 1.792 mm and the mean distance between the K2 wire, and the posterior cortex was 20.750 ± 1.473 mm, mean ratio 0.8083 ± 0.081 (Fig. 7a).

**Fig. 8:**
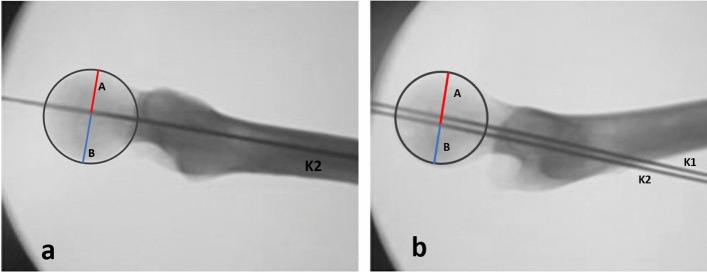
Through Brainlab-TraumaCad® software image femoral head was normalized by circles passing through the apex, the anterior extremity and the extreme posterior of the femoral head cortex in both Löwenstein Lateral view (LLV) and True Lateral view (TLV). **a** On the image obtained through the TLV, K1 and K2 are superimposed. We drew two lines perpendicular to the second Kirschner (K2) and passing through the center of the circumference obtained from the normalized femoral head, thus measuring the distance between K2 and the anterior cortex (A) and the distance between K2 and posterior cortex (B). **b** On the image obtained through the Löwenstein Lateral view (LLV) we drew two lines perpendicular to the second Kirschner wire (K2) and passing through the center of the circumference obtained from the normalized femoral head, thus measuring the distance between K2 and the anterior cortex (A) and the distance between K2 and posterior cortex (B)

On the LLV the distance between the K2 wire to the anterior and posterior cortex of the femoral head was 24.362 ± 1.085 mm and 19.650 ± 1.531 mm, respectively, ratio 1.2486 ± 0.136 (Fig. 8).

On the image of the dissected femoral head the distance of K1 wire from the superior femoral head cortex was 19.112 ± 1.701 mm, while from the inferior cortex was 20.937 ± 1.595 mm, ratio 0.9138 ± 0.038; the distance of K2 wire from the superior femoral head cortex was 9.5875 ± 0.916 mm, while from the inferior cortex was 30.412 ± 1.493 mm, ratio 0.3164 ± 0.039.

**Fig. 9:**
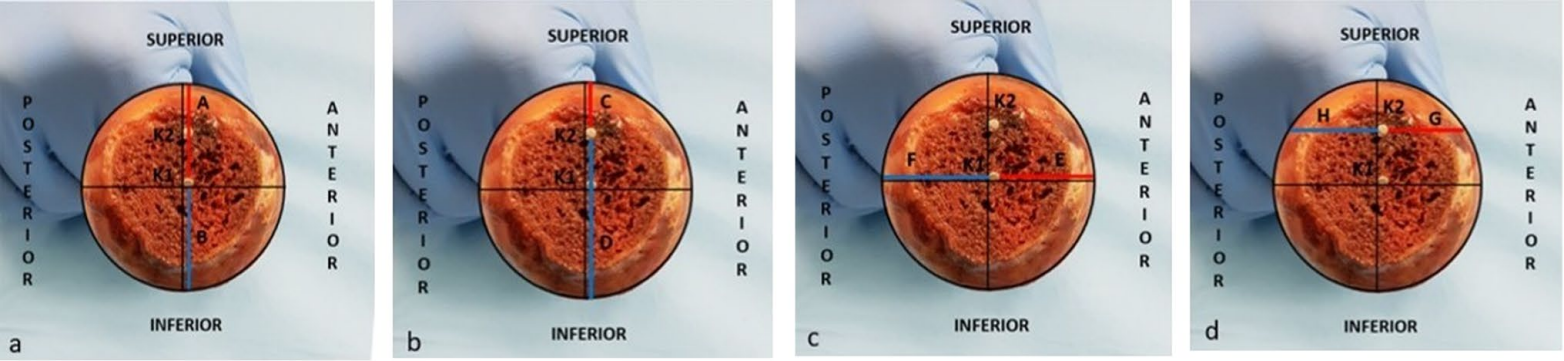
On the image of the dissected femoral head, through the Brainlab-TraumaCad® software we normalize femoral head measure on photographs by circles passing through the superior cortex, the anterior extremity, and the extreme posterior of the femoral head cortex. **a** We drew two lines perpendicular to the first Kirschner wire (K1) and to the diameter of the circumference obtained from the normalized femoral head, thus measuring the distance between K1 and the superior cortex (A) and the distance between K1 and inferior cortex (B).** b** We drew two lines perpendicular to the second Kirschner wire (K2) and to the diameter of the circumference obtained from the normalized femoral head, thus measuring the distance between K2 and the superior cortex (C) and the distance between K2 and inferior cortex (D).** c** We drew two lines perpendicular to the K1 and to the diameter of the circumference obtained from the normalized femoral head, thus measuring the distance between K1 and the anterior cortex (E) and the distance between K1 and posterior cortex (F).** d** We drew two lines perpendicular to the K2 and to the diameter of the circumference obtained from the normalized femoral head, thus measuring the distance between K2 and the anterior cortex (G) and the distance between K2 and posterior cortex (H)

The distance of K1 wire from the anterior cortex was 19.287 ± 1.652 mm, while from the posterior cortex was 20.712 ± 1.278 mm, ratio 0.9304 ± 0.038. The distance between K2 wire and the anterior cortex was 15.912 ± 1.481 mm, while the distance between K2 wire and the posterior cortex was 17.637 ± 1.329 mm, ratio 0.9020 ± 0.046 (Fig. 9).

The accuracy of the AP view was 0.9705 (Δ = 0.0295) for the K1 and 0.9930 (Δ = 0.007) for the K2.

The accuracy of the TLV and LLV was 1.1479 (Δ = 0.1479) and 1.1584 (Δ = 0.1584), respectively, when the wire was positioned at the center of the head on the AP view and TLV (K1). When the wire was positioned proximal in AP view and central in TLV (K2) the accuracy of the TLV and LLV was 1.1159 (Δ = 0.1159) and 0.7224 (Δ = 0.2776), respectively. The highest accuracy was found for K2 on the AP view (Δ = 0.007), while the worst accuracy was revealed for K2 on the LLV (Δ = 0.2776). The results are summarized in Table 2.

**Table 2:** The accuracy and Δ of the AP view, Löwenstein lateral view (LLV) and True lateral view (TLV)

	K1				K2			
	True values	Measured values	Accuracy	Δ	True values	Measured values	Accuracy	Δ
AP	0.9138	0.9416	0.9705	**0.0295**	0.3164	0.3186	0.9930	**0.007**
TLV	0.9304	0.8105	1.1479	**0.1479**	0.9020	0.8083	1.1159	**0.1159**
LLV	0.9304	0.8032	1.1584	**0.1584**	0.9020	1.2486	0.7224	**0.2776**

Most important values are in given in bold

## Discussion

There is currently a lack of systematic research, with no real gold standard on intraoperative fluoroscopy of pertrochanteric fractures. A large number of surgeons are not educated on how to assess exactly intraoperative imaging of the proximal femur [15]. Our study aims to help orthopedic surgeons on the interpretation of intraoperative radiographic projections and, therefore, in making the correct choice.

Cut-out is defined as a varus collapse of the femoral head with associated protrusion of the cephalic screw from the femoral head itself [11, 16]. This is the main cause of mechanical failure in both intramedullary and extramedullary cephalic implants for pertrochanteric fractures fixation [17]. Several variables are associated to cut-out, but the Tip-Apex Distance [8] still remains the main predictor [18]. Hence, the importance of an optimal position of the cephalic screw resulting from a correct interpretation of intraoperative radiographic images.

In our study the accuracy calculated on the AP view was comparable for the K1 and K2 wires (0.9705 and 0.9930, respectively) with values very close to a maximus of 1. Therefore, the accuracy was very high for both K1 and K2, indicating that the position of the two wires in this projection was very close to reality.

Varying the inclination of the C-arm from the TLV to the LLV, we observed no modification of the central K-wire (K1) position and a change in position of the K-wire proximally located (K2) (Fig. 4b, c). Comparing the accuracy of the LLV between K1 and K2 wire, we noticed a lower accuracy for K2 wire (0.7224) compared to K1 (1.1584). Positioning the K-wire at the center of the head in AP view and in TLV (K1), the accuracy of the TLV and LLV appears completely similar (1.1479 and 1.1584, respectively) with values very close to maximus value of 1. Therefore, given the high accuracy, when the K-wire is positioned in the center of the neck/head, its position is real in both projections.

When the wire was positioned proximal to the central one on AP view and central in TLV (K2) the accuracy of the TLV and LLV projection appears to be different (1.1159 and 0.7224, respectively). In this case, the TLV was, in fact, the projection closer to the maximum value of 1 and therefore the most accurate. Thus, the location of the K-wire on the LLV deviates from its real position.

The LLV does not consider the anteversion of the femoral neck and therefore should not be considered a true lateral projection, but rather an oblique view. Assuming that femoral head is a sphere, the center of the head is the same in all the projections considered (LLV, TLV and AP view). However, LLV does not allow for the accurate determination of the anterior or posterior position of the K-wire (or cephalic screw) in case of eccentric positioning.

The TLV considers the femoral neck anteversion and is therefore perfectly orthogonal to the AP view. This allows for the quantification of the real degree of AP deviation of the K-wire without being influenced by the K-wire position on the AP view itself.

Considering the relationship between the implant position and the risk of complication, there is nowadays a wide introduction of computer-assistance and robotic-assistance of proximal femoral nailing [19–25]. Muramaki et al., using the ADAPT system (Stryker Kalamazoo, Michigan, USA), compared the intraoperative Tip-Apex Distance and Tip to Head Surface Distance with the relative CT postoperative measures, and demonstrated the high accuracy of the system. In the systematic review performed by Li et al. [23] it was well established that the computer-assistance systems improve the mean Tip-Apex Distance compared to free-hand lag screw positioning, but there is no decreasing of surgical time and radiation time. Similarly, Coviello et al. [22], which evaluated ATLAS system (Masmec Biomed, Modugno, Bari, Italy), found a longer room set-up time compared to traditional nailing. Therefore, we believe that the cost–benefit of the computer-assistance systems need to be better established in future, and the correct intraoperative radiological analysis remains essential in hip osteosynthesis.

The importance of fluoroscopic imaging of the proximal femur during an antegrade nailing is not only crucial for screw positioning but also for achieving a satisfying reduction, as demonstrated by Chen et al. [26]. They found the relevance of a 30° oblique tangential projection to study the anteromedial cortex, which is considered a keystone in the proximal femur. Contact cortex-to-cortex is essential to provide a buttress function to the neck-head complex.

Another surgical technique for hip fracture is femoral neck screwing. In this technique as well, the fluoroscopic intraoperative evaluation is important and it is usually based on an “inverted triangle” configuration of the screws [27]. One possible complication is “in–out-in” positioning of the posterosuperior screw [28]. Hoffmann et al. demonstrated that 70% of the posterosuperior screws are placed as “in–out-in” even though they may appear inside the bone in antero-posterior and lateral views [27]. This carries the risk of osteonecrosis due to lateral epiphyseal artery [28, 29], and less mechanical strength of the construct [30, 31]. Therefore, we believe that a thorough and reliable fluoroscopic intraoperative assessment is mandatory to achieve a correct reduction and fixation.

By positioning the K-wire at the center of the neck/head, all the projections used (AP, TLV, LLV) showed high accuracy and accurately represented the true position of the wire. When the K-wire was placed proximally in AP view and centrally in TLV (K2), only the TLV showed high accuracy and expressing the real position of the K-wire. However, as we observed, these considerations about the TLV were applicable only if the K-wire was placed parallel to the diaphysis and the femoral neck. If the parallelism was not respected, the TLV lost accuracy.

Our study had some technical limitations that need to be considered. Not using the whole inferior limb specimen may be considered a limit, because only the femur could not re-create the real-time situation of the operating room. Femoral head is not perfectly spherical, and the femoral neck is not perfectly cylindrical. Even in a cadaveric study, it is difficult to place the K-wire perfectly in the center of the head and in the planned positions. These were the reasons why we could not achieve an accuracy exactly equal to 1. To simplify the study, we decided to use only two K-wires. We hypothesize that the same considerations can also be applied to any eccentric position (proximal, distal, medial, lateral) of the K-wire, provided that parallelism with the neck and shaft of femur is maintained.

## Conclusions

In conclusion, by positioning the K-wire at the center of the neck/head, all the projections used (AP, TLV, LLV) show high accuracy expressing the true position of the wire. When the K-wire was positioned proximal in AP view and central in TLV, *only the TLV showed high accuracy and represented the real position of the K-wire*; this because TLV is perfectly orthogonal to the AP view allowing to quantify the real degree of AP deviation of the K-wire.

## Data Availability

All data are available in the main text and tables. Additional information can be provided if solicited.
